# Characterization and anti-inflammation role of swine IFITM3 gene

**DOI:** 10.18632/oncotarget.20568

**Published:** 2017-08-27

**Authors:** He-Ping Li, Pei-Ge Chen, Fu-Tao Liu, He-Shui Zhu, Xian-Qin Jiao, Kai Zhong, Yu-Jie Guo, Guang-Ming Zha, Li-Qiang Han, Wei-Fei Lu, Yue-Ying Wang, Guo-Yu Yang

**Affiliations:** ^1^ Key Laboratory of Animal Biochemistry and Nutrition, Ministry of Agriculture, Henan Agricultural University, Zhengzhou, Henan, China

**Keywords:** IFITM3, characterization, anti-inflammation, TLR4 signaling pathway, lipopolysaccharide, Immunology and Microbiology Section, Immune response, Immunity

## Abstract

IFITM3 is involved in cell adhesion, apoptosis, immune, and antivirus activity. Furthermore, IFITM3 gene has been considered as a preferential marker for inflammatory diseases, and positive correlation to pathological grades. Therefore, we assumed that IFITM3 was regulated by different signal pathways. To better understand IFITM3 function in inflammatory response, we cloned swine IFITM3 gene, and detected IFITM3 distribution in tissues, as well as characterized this gene. Results indicated that the length of swine IFITM3 gene was 438 bp, encoding 145 amino acids. IFITM3 gene expression abundance was higher in spleen and lungs. Moreover, we next constructed the eukaryotic expression vector PBIFM3 and transfected into PK15 cells, finally obtained swine IFITM3 gene stable expression cell line. Meanwhile, we explored the effects of LPS on swine IFITM3 expression. Results showed that LPS increased IFITM3 mRNA abundance and exhibited time-dependent effect for LPS treatment. To further demonstrate the mechanism that IFITM3 regulated type I IFNs production, we also detected the important molecules expression of TLR4 signaling pathway. In transfected and non-transfected IFITM3 PK15 cells, LPS exacerbated the relative expression of TLR4-NFκB signaling molecules. However, the IFITM3 overexpression suppressed the inflammatory development of PK15 cells. In conclusion, these data indicated that the overexpression of swine IFITM3 could decrease the inflammatory response through TLR4 signaling pathway, and participate in type I interferon production. These findings may lead to an improved understanding of the biological function of IFITM3 in inflammation.

## INTRODUCTION

Swine diseases have disturbed many intensive farms heavily in economic and social losses, and aroused extensive attention to the prevention and treatment of swine diseases. Due to the complex genetic diversity, current vaccination and antiviral strategies are not valid [1]. The innate immune is the first line for host defense against infections. As one type of potent innate immunomodulators, interferons (IFNs) exert an important role in the host against infection and pathogen invasion via several diverse mechanisms. IFNs can inhibit a variety of viruses, bacteria, and parasites infections [2].

Interferon inducible trans-membrane protein 3 (IFITM3) is a double trans-membrane protein that belongs to the IFITM family members. IFITM3 is mainly stimulated by IFNs participating in various biological processes. Hence, IFITM3 is involved in interferon-triggered processes, such as anti-proliferative activities of different pathogenesis, antivirus infections in innate immune response, cell adhesion, cell apoptosis, and germ cell homing [2-10]. More importantly, IFITM3 is also a potent antiviral and anti-inflammatory effector in the host innate immune system [7, 8, 11]. At present, the characteristics and antiviral activity of human IFITM3 have been broadly investigated, and mainly focused on human-associated pathogens.

Currently, studies on swine IFITM3, especially regarding its function, are very few. Although previous studies on IFITMs mainly focused on their roles in embryonic development, their functions as host antiviral factors were only recently discovered by RNA interference genomic screening for host factors involved in influenza virus infection [7]. It was subsequently revealed that IFITMs can restrict the early stages of replication for a wide variety of viruses, including Influenza virus, West Nile viruses, Ebola viruses, and Corona virus [7, 8, 12]. Researchers found that IFITM3 gene was mainly isolated from severely inflamed mucosa and considered as a biomarker for ulcerative colitis [6, 13]. Furthermore, IFITM3 expression increased in gastric cancer, and colorectal tumors [14-16]. There exists a positive correlation between the IFITM3 expression levels and the pathological glioma grades [17]. Previous studies have shown that toll like receptor 4 (TLR4) may play a major role in various inflammatory diseases. Literatures demonstrated that TLR4 expression up-regulated in rheumatoid arthritis, systemic lupus erythematosus, and multiple sclerosis [18-23]. Bacterial lipopolysaccharide (LPS), which is recognized by host innate immune receptor TLR4 and subsequently triggers inflammation by activation of a transcriptional factor IFN regulatory factor and nuclear factor-κB (NFκB), resulting in up-regulation of various inflammatory mediators [24].

However, the role of swine IFITM3 in inflammation and the regulatory signal pathways is unclear. In this study, we first cloned swine IFITM3 gene from spleen tissues and examined swine IFITM3 mRNA location in tissues. To better understand IFITM3 function in inflammation, we next constructed the eukaryotic expression vector PBIFM3 and transfected into PK15 cells, as well as obtained swine IFITM3 gene stable expression cell line. Meanwhile, we explored the effects of LPS on swine IFITM3 expression. We further also detected the important molecules expression of TLR4 signaling pathway. Together, these data indicated that overexpression of swine IFITM3 could decrease the inflammatory response through TLR4 signaling pathway, and participate in type I interferon production. These conclusions may be helpful to understand deeply the biological function of IFITM3 in inflammation.

## RESULTS

### Characterization and expression pattern of IFITM3 gene

**Figure 1:**
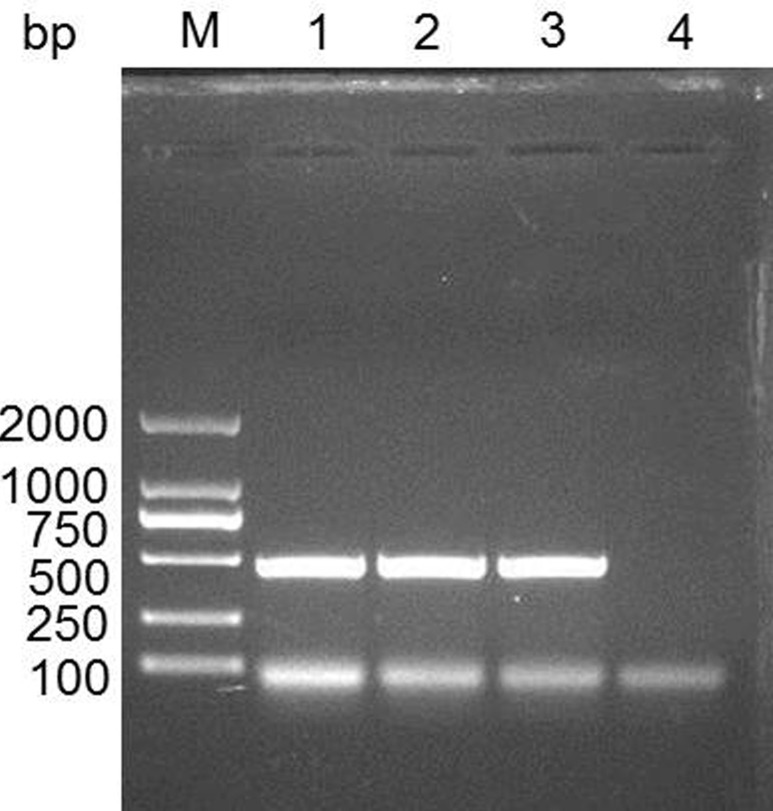
Cloning the IFITM3 gene in swine spleen tissue M, DL2000 DNA marker; 1-3, PCR product for swine IFITM3; 4, negative control.

We searched the predicted IFITM3 CDS (NM_001201382.1) in the NCBI database. The length of IFITM3 CDS is 438 bp, and encodes 145 amino acids. We cloned IFITM3 CDS and identified it using DNA sequencing. The data demonstrated the expected nucleotide sequence of IFITM3 as NCBI database described (Figure 1).

**Figure 2:**
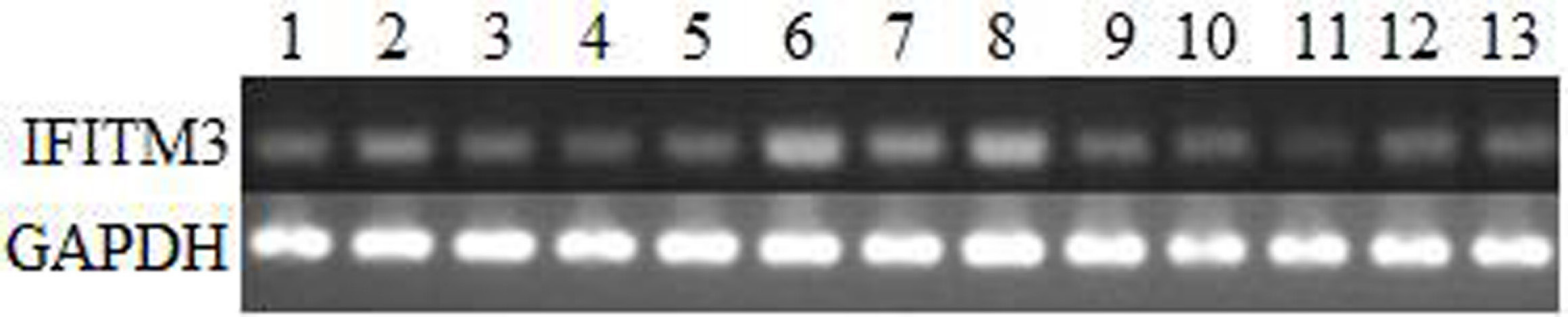
Expression patterns of the IFITM3 mRNA in various swine tissues 1, skin; 2, muscle; 3, adipose; 4, heart; 5, liver; 6, spleen; 7, kidney; 8, lung; 9, duodenum; 10, jejunum; 11, ileum; 12, cecum; 13, rectum.

To illustrate the expression patterns of IFITM3 mRNA in tissues, we detected IFITM3 mRNA expression in 13 healthy swine tissues (Figure 2). Results showed that IFITM3 mRNA was expressed in all 13 tissues. Moreover, the IFITM3 mRNA abundance was relatively higher in spleen and lung, while the IFITM3 mRNA in heart and ileum was hardly detected (Figure 2). These differential expression implicated that IFITM3 might be involved in the functional regulation of immunity.

### Transfection efficiency and IFITM3 overexpression

**Figure 3:**
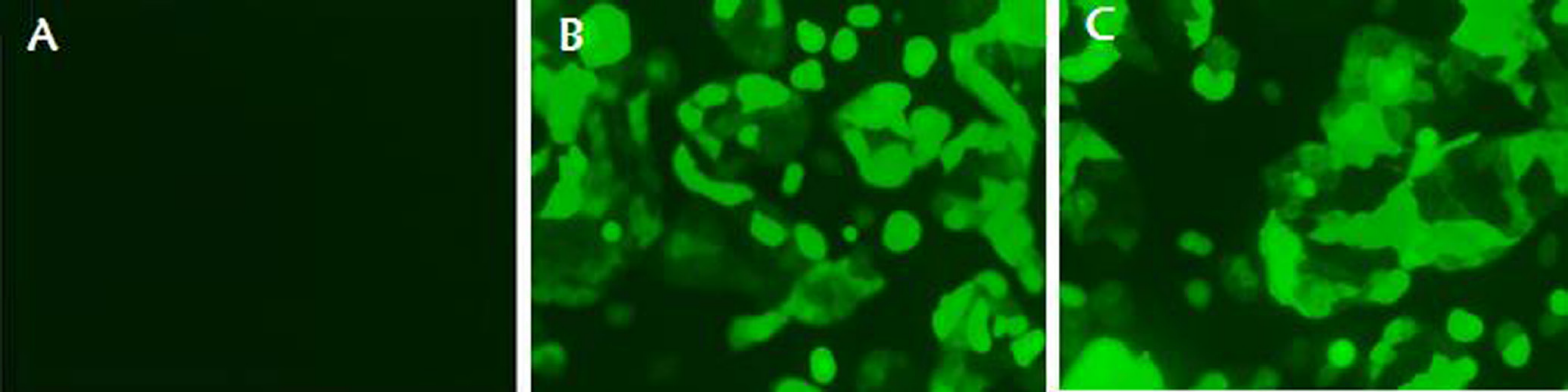
Observation of green fluorescence in PK15 cells transfected with IFITM3 expression vectors Green fluorescence was observed clearly using fluorescence microscopy in the PBv (**B**), and PBIFM3 (**C**) cells, but not in non-transfected PK15 cells (**A**).

**Figure 4:**
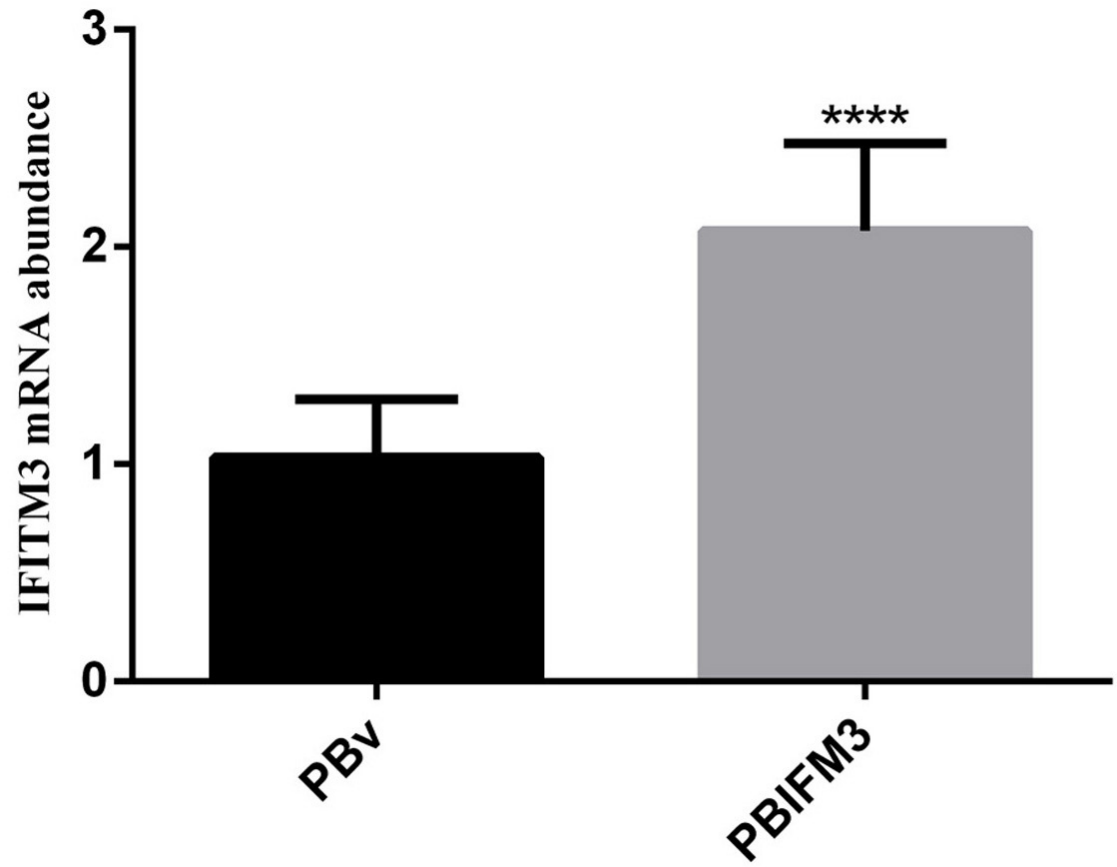
Overexpression of the swine IFITM3 gene in PBIFM3 cells GAPDH was used as an internal housekeeping gene. **** *P* < 0.0001 vs. PBv.

To further evaluate the IFITM3 function *in vitro*, we next constructed the eukaryotic expression vector PBIFM3 and transfected into PK15 cells, as well as obtained swine IFITM3 stable expression cell line. Based on Piggyback (PB) vector carries the green fluorescent protein (GFP) gene. When the PB vector is integrated into the host cell genome, GFP protein is expressed in the host cells, which can be seen under a fluorescence microscope. We clearly observed the green fluorescence in the transfected PBIFM3 and PBv, but not in the non-transfected PK15 cells (Figure 3). These results indicated that vectors with the GFP gene were successfully transfected into cells and integrated into the genome. Through screening, we obtained stable transfected cell lines. To evaluate IFITM3 expression efficiency, we further detected the expression of IFITM3 using real-time RT-PCR, combined with observation under microscope (Figure 3). The IFITM3 mRNA expression up-regulated significantly in PBIFM3 cells, compared with that in PBv cells (*p* < 0.0001) (Figure 4). Thus, IFITM3 was successfully transfected into PK15 cells and efficiently overexpressed.

### IFITM3 expression in inflammatory response

**Figure 5:**
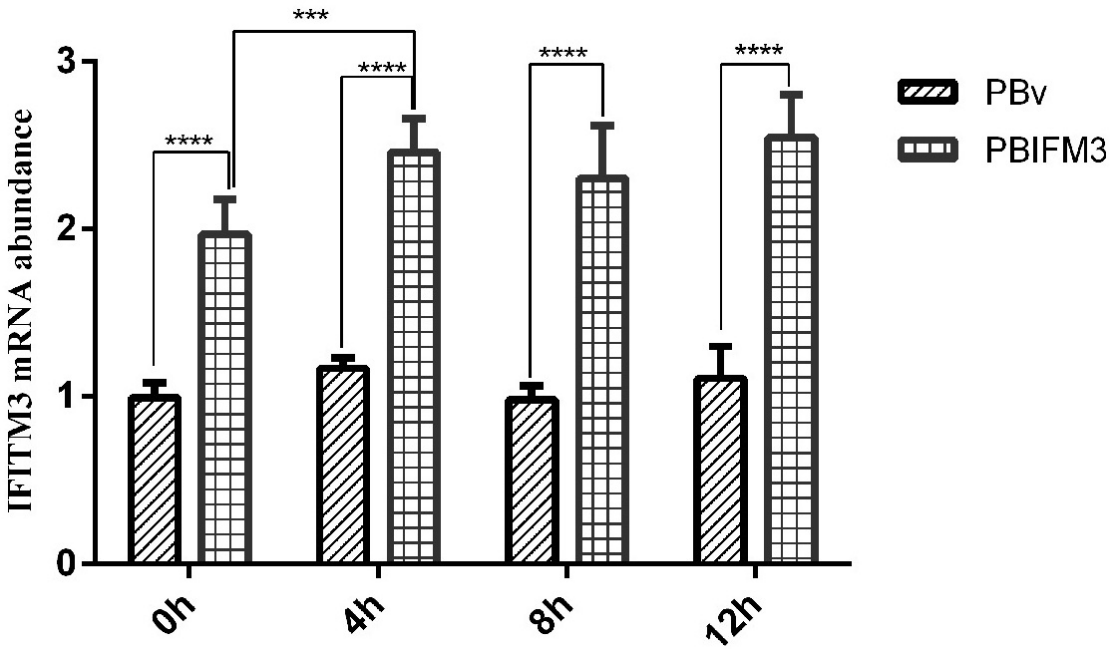
Effect of LPS on swine IFITM3 expression Graphs demonstrate the expression of IFITM3 in PBIFM3 and PBv cells stimulated with or without LPS (100 μg/ml). In LPS groups, cells were incubated correspondingly with LPS (100 μg/mL) for 4, 8, 12 h. Cells in 0 h (control) group were cultured in basal medium. ****P < 0.0001 vs. PBv or 0 h group.

LPS is the most commonly used experimental inflammatory factor. We treated the PBIFM3 and PBv cells with or without LPS to detect IFITM3 expression in inflammatory response. As shown in Figure 5, compared with 0 h cells, LPS increased IFITM3 mRNA abundance in PBIFM3 and PBv, and exhibited the time-dependent effect for LPS treatment. However, IFITM3 mRNA abundance in PBIFM3 cells was notably higher than that in PBv cells (*p* < 0.0001). These data noted that the IFITM3 expression can be up-regulated in inflammatory response.

### IFITM3 inhibits type I interferons production

**Figure 6:**
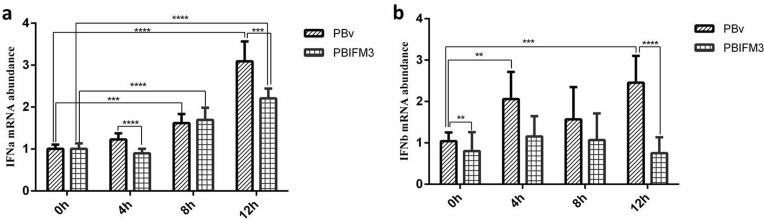
Effect of swine IFITM3 on the expression of IFNα and IFNβ Graphs demonstrate the expression of IFNα (**a**) and IFNβ (**b**) in PBIFM3 and PBv cells stimulated with or without LPS (100 μg/ml). In LPS groups, cells were incubated correspondingly with LPS (100 μg/mL) for 4, 8, 12 h. Cells in 0 h (control) group were cultured in basal medium. **P < 0.01, ***P < 0.001, ****P < 0.0001 vs. PBv or 0 h group.

Type I interferons are critical for blocking pathogens infection by promoting hundreds of IFN-stimulated genes production [25]. IFITM3 gene has been identified in several cellular processes mediated by IFNs, such as antivirus, cell adhesion and anti-proliferative activities [3]. We detected type I interferons production in PBIFM3 cells with or without LPS treatment. As shown in Figure 6, IFNɑ was significantly up-regulated in the PBIFM3 and PBv cells, as well as dependent on the time of LPS treatment. However, IFNɑ abundance in PBIFM3 cells was lower than that in PBv cells. Similar results were also observed in the change of IFNβ expression, IFNβ expression was not entirely dependent on the time of LPS treatment. Results implicated that IFITM3 decreased the IFNs production during inflammatory activity.

### IFITM3 suppresses TLR4 signaling pathway

**Figure 7:**
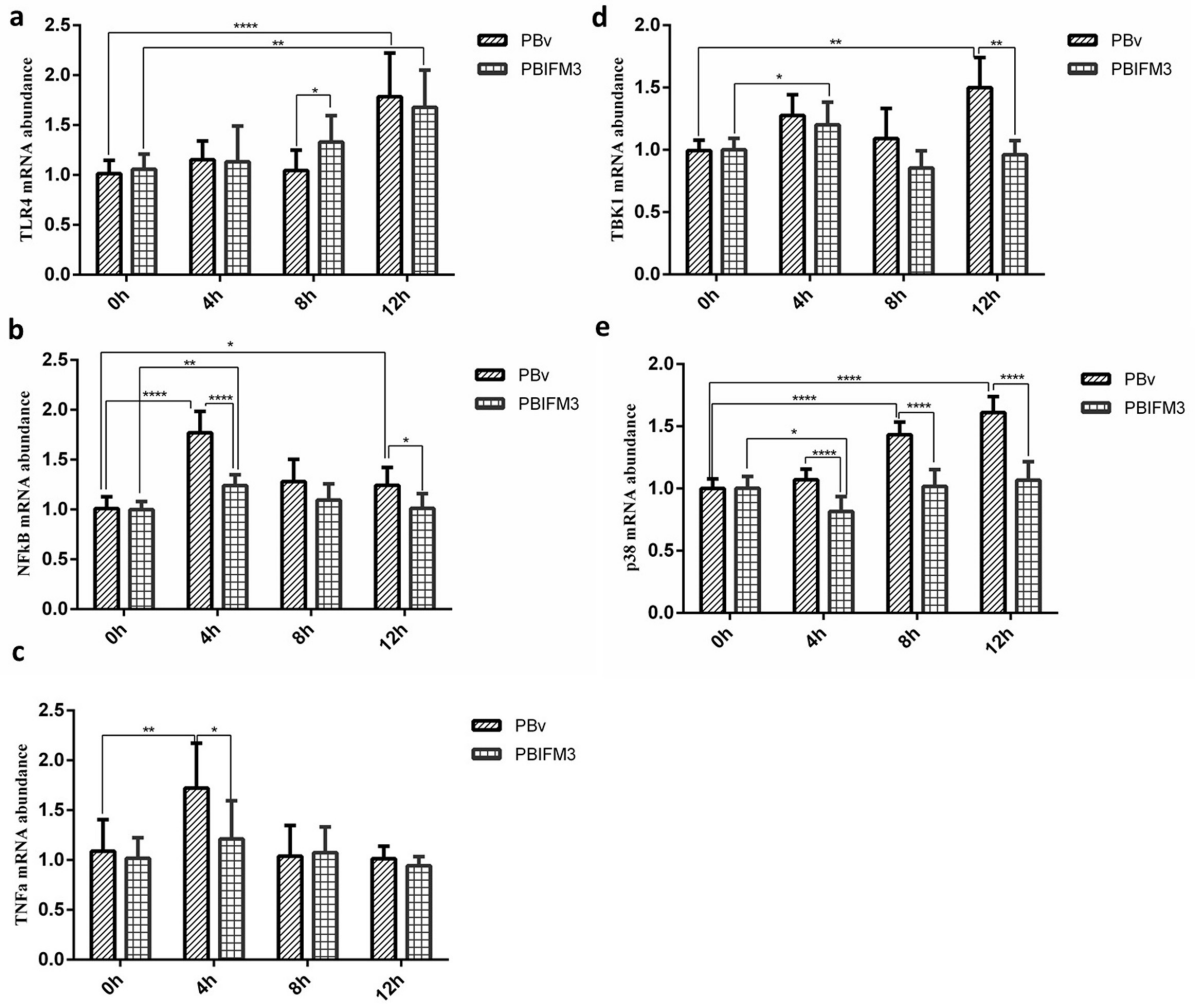
Effect of swine IFITM3 on the expression of components of the TLR4 signaling pathway Graphs demonstrate the expression of TLR4 (**a**), NFκB (**b**), TNFα (**c**), TBK1 (**d**) and p38 (**e**) in PBIFM3 and PBv cells stimulated with or without LPS (100 μg/ml). In LPS groups, cells were incubated correspondingly with LPS (100 μg/mL) for 4, 8, 12 h. Cells in 0 h (control) group were cultured in basal medium. *P < 0.05,**P < 0.01, ****P < 0.0001 vs. PBv or 0 h group.

To further clarify the mechanism that IFITM3 regulated type I IFNs production, we also detected the important molecules expression of TLR4 signaling pathway, which is the classical pathway of LPS. In transfected and non-transfected IFITM3 PK15 cells, LPS exacerbated the relative expression of TLR4-NFκB signaling key molecules (Figure 7), and up-regulated the expression of TLR4, NFκB, p38 mitogen-activated protein kinases (p38 MAPK), and tumor necrosis factor alpha (TNFα), compared with 0 h treatment cultured with basal medium only (Figure 7). However, the IFITM3 overexpression suppressed the inflammatory development of PK15 cells (Figure 7), and down-regulated the expression of TLR4, NFκB, p38 MAPK, and TNFα, compared with the PBv cells treated with LPS (Figure 7). Consistently, the levels of TNFα and NFκB reached at peak 4 h after LPS treatment both of the PBIFM3 and PBv cells, then decreased over time. The relative expression of TLR4 and p38 MAPK were dependent on the time of LPS treatment. However, the change of TANK-binding kinase 1 (TBK1) expression had no significance correlation with LPS treatment time. Collectively, these results indicated that IFITM3 was involved in the TLR4-NFκB signaling pathway and played a pivotal role in inflammatory response.

## DISCUSSION

IFITM proteins are approximately 130 amino acids in length and are conserved in most vertebrate species [3]. Xu et al reported that the full-length cDNA of swine IFITM3 from lymph node tissue is 438 base pairs (bp), and encodes 145 amino acids residues [26]. In this study, we cloned swine IFITM3 gene, and detected the IFITM3 distribution in tissues, as well as functional characterized this gene. The results similar with Xu et al [26] were also observed. The length of swine IFITM3 gene was also 438 bp, encoding 145 amino acids. According to the gene distribution in tissues, IFITM3 gene expression abundance is higher in spleen and lungs.

IFITM3 has been reported to participate in adhesion, apoptosis, immune response, growth and development, and germ cell homing [3]. More importantly, IFITM3 exhibits the greatest protection against the broadest range of viruses, including influenza A virus, flaviviruses, hepaciviruses, and reoviruses, etc [11, 12, 25, 27]. IFITM3 blocked human influenza virus infection during the early stages of virus entry, precisely, after hemifusion, likely due to the relatively high constitutive expression in the endosomal and lysosomal of host [28]. Furthermore, the single-nucleotide polymorphism of IFITM3 gene resulted in decreasing IFITM3 protein expression, which has been linked with a higher risk of virus infections [29, 30]. IFITM3 knockout mice increased morbidity and mortality associated with seasonal or pandemic influenza virus infection [29, 31]. Recent studies identified that IFITM3 gene was a powerful biomarker for ulcerative colitis [6, 13]. Moreover, IFITM3 up-regulated in gastric cancer, and colorectal tumors [14-16]. Furthermore, study showed that there existed a positive correlation between the IFITM3 expression levels and pathological grades [17]. In this study, the IFITM3 mRNA abundance was hardly detected in ileum (Figure 2). We considered that different intestine and pathological grades may be the major reason resulting in the differential result. However, some studies indicated that IFITM3 was necessary to regulate the complex IFN-α response [32, 33]. Therefore, we assumed that IFITM3 could be regulated by several signal pathways.

As we all know, TLR4-NFκB pathway participates in various inflammatory cytokines production, which is classical pathway of LPS, and subsequently triggers inflammation by activation of a transcriptional factor IFN regulatory factor and NFκB, resulting in the up-regulation of various inflammatory mediators [24]. To better understand IFITM3 function in inflammatory response, we transfected PBIFM3 vector into PK15 cells to achieve IFITM3 overexpression. PB is a DNA transposon which was originally isolated from genomes of baculoviruses that infect cabbage looper moth Trichoplusiani [34]. PB vector uses the TTAA sequence to insert into target sites, and displays little selectivity for particular regions of the genome other than a modest preference for DNase I sensitivity regions [35, 36]. Others have shown that PB is distinguished by its ability to remain active when fused to a DNA binding domain and that such fusions can bias insertion toward cognate sites [37, 38, 39]. Hence, we used PB vector and constructed the eukaryotic expression vector PBIFM3, and then transfected into PK15 cells, as well as obtained swine IFITM3 stable expression cell line. Meanwhile, we explored the effects of LPS on swine IFITM3 expression. Results showed that LPS increased IFITM3 mRNA abundance and exhibited the time-dependent effect for LPS treatment.

TLR4-NFκB signaling has been reported to play a critical role in the LPS-induced expression of inflammatory cytokines such as TNFα and interleukin 1 beta (IL-1β) in macrophages and gingival fibroblasts [40, 41]. p38 MAPK belongs to a class of serine/threonine kinases and expresses in most tissues [42]. p38 MAPK activates a wide range of transcription factors, protein kinases, cytosolic and nuclear proteins, all of which lead to diverse responses such as inflammation, cell differentiation, apoptosis, and cytokine production [43]. TBK1 plays important roles in innate immunity. TBK1 mediates the activation of interferon regulatory factor, leading to the induction of type I IFNs [44]. Furthermore, Nakajima et al has shown that the TBK1, p38 MAPK, and NFκB pathways are involved in the IFITM3 expression induced by LPS [45]. We detected type I interferon production in PBIFM3 cells with or without LPS treatment. As shown in Figure 6, IFITM3 down-regulated IFNs production during inflammatory activity. To further illustrate the mechanism that IFITM3 regulated type I IFNs production, we next detected the important molecules expression of TLR4 signaling pathway. In transfected and non-transfected IFITM3 PK15 cells, LPS aggravated the relative expression of TLR4-NFκB signaling molecules (Figure 7). However, the IFITM3 overexpression suppressed the inflammatory development of PK15 cells (Figure 7).

In summary, these data indicated that the overexpression of swine IFITM3 could decrease the inflammatory response through TLR4 signaling pathway, and participate in type I interferon production. These findings may lead to an improved understanding of the biological function of IFITM3 in inflammation.

## MATERIALS AND METHODS

### Reagents

LPS (*E. coli* O55:B5), and puromycin were bought from Sigma (St Louis, MO, USA). Dulbecco’s modified Eagle’s medium (DMEM) and fetal bovine serum (FBS) were obtained from Gibco (Life Technologies, Carlsbad, CA, USA). Trizol reagent, Prime Script RT reagent kit, SYBR Premix Ex Taq, and pMD19-T were purchased from TaKaRa Bio Inc. (Shiga, Japan). PB vector and PB transposes were obtained from SBI Ltd. (Palo Alto, CA, US). Other reagents were purchased from Sino Pharm Chemical Reagent Ltd. (Shanghai, China).

### Tissues samples

A total of 13 tissues samples including skin, muscle, fat, heart, liver, spleen, kidney, lung, dudens, jejunum, ileum, cecum, rectum from 5 healthy swines (Crossbred: Duroc × Landrace × Yorkshire) were collected, washed three times in phosphate buffered saline (PBS, pH 7.2) and immediately snap-frozen in liquid nitrogen before being stored at -80^◦^C for further use.

### Cloning IFITM3 gene

Swine IFITM3 genomic sequence (NM_001201382.1) was searched in the GenBank database. Using this sequence, a pair of primers (F: 5’-ACTGTCGACAT GAACTGCGCTTCCCAGCCCTTC-3’; R: 5’-ACTGCGGCCGCGTAGCCTCTGT AATCCTTTATG-3’) containing the *Sal I* and *Not I* (TaKaRa, Shiga, Japan) restriction sites were designed. Total RNA was extracted from swine tissues using Trizol reagent (TaKaRa, Shiga, Japan), and RNA was then used for cDNA synthesis. IFITM3 fragment was amplified; the final PCR product was isolated by electrophoresis using 1 % polyacrylamide gels, and purified using an agarose gel extraction kit. The purified target fragment was ligated into the plasmid pMD19-T and then transformed into competent *E.coli DH5a* cells. Recombinant plasmid was extracted from bacterial colonies and plasmid solution was subjected to agarose gel electrophoresis to confirm the presence of the correct sequence of IFITM3. The length of the amplification segment was 438 bp.

### Expression pattern of IFITM3 mRNA in tissues

Total RNA was prepared from the snap-frozen tissue using Trizol reagent and real-time RT-PCR was adopted to detect the expression of IFITM3 mRNA in different tissues. The relative abundance of IFITM3 mRNA was evaluated by the melt cycle threshold method and standarded with swine GAPDH mRNA level as an inter reference with specific primers for GAPDH (listed in Table 1).

**Table 1:** Primers used for RT-PCR amplification

Gene	Primers	Length (bp)	Access No.
IFITM3	F: 5’- TGGTGGGAGACATCATTGGG-3’ R: 5’- GAAAATTACCAGGGAGCCAGTG-3’	134	NM_001201382.1
IFNα	F: 5’- TGGTGCATGAGATGCTCCA-3’ R: 5’- GCCGAGCCCTCTGTGCT-3’	55	XM_021062981.1
IFNβ	F: 5’- TCGCTCTCCTGATGTGTTTC-3’ R: 5’- TTCTGACATGCCAAATTGCT-3’	94	NM_001003923.1
p38	F: 5’- CTTACGGATGACCACGTTCAGT-3’ R: 5’- GCTCACAGTCTTCATTCACAGC-3’	127	XM_005318767.3
TNFα	F: 5’- AACCCTCTGGCCCAAGGA-3’ R: 5’- GGCGACGGGCTTATCTGA-3’	57	NM_214022.1
TBK1	F: 5’- ACAGATTTTGGTGCAGCCAG-3’ R: 5’- CCTTATTCCTACGTGGCCCT-3’	229	XM_021090854.1
NFκB	F: 5’- CCCATGTAGACAGCACCACCTATGAT-3’ R: 5’- ACAGAGGCTCAAAGTTCTCCACCA-3’	132	NM_001048232.1
TLR4	F: 5’- TCAGTTCTCACCTTCCTCCTG-3’ R: 5’- GTTCATTCCTCACCCAGTCTTC-3’	166	NM_001293316.1
GAPDH	F: 5’- TGGTGAAGGTCGGAGTGAAC-3’ R: 5’- GGAAGATGGTGATGGCCTTTC-3’	369	XM_021091114.1

### Vectors constructions

Construction of the eukaryotic expression vector PB-IFITM3 was as follows. IFITM3 cDNA (438 bp length) was subcloned from pMD19-T-IFITM3 into the PB vector (that contained GFP and puromycin resistance genes) after double restriction enzyme digestion. The obtained recombinant expression vector was called PB-IFITM3. *Nhe* I and *EcoR* I were used to digest PB-IFITM3, and then identified them through agarose gel electrophoresis. Sequencing using both forward and reverse primers was performed to confirm the above recombinant vectors.

### Cell culture, transfection, and photograph

PK15 porcine kidney epithelial cells were maintained in DMEM medium including 10 % FBS (v/v) and 1 % penicillin/streptomycin (v/v), and cultured at 37°C with 5 % CO_2_ (MCO-5AC CO_2_ Incubator, Sanyo, Tokyo, Japan). Prior to transfection experiments, PK15 cells were plated at the density 2×10^5^ cells per well in six-well plates. The PK15 cells were transfected with PB-IFITM3 and correspondingly in parallel with empty vector, using lipofectamine 2000 reagent (Life Technologies), according the manufacturer’s protocol. Based on transfecting plasmids, cells were divided into three groups: PB-IFITM3, PB-vector and non-transfection. After 12 h transfecting, the images were captured using DAS microscope Leitz DM RB with a dual mode cooled charged coupled device (CCD) camera (C4880; Hamamatsu, Japan) to observe GFP expression. The transfected cells were selected for on 5 μg/mL puromycin for 2 weeks. Cells that were not transfected with the target gene were killed, and we achieved stable transfection cell lines, named PK15-PB-IFITM3 (PBIFM3), and PK15-PB vector (PBv).

### Transfection efficiency and overexpression detection

The PB vector carries both the purine gene and the GFP gene. Therefore, when the PB vector was transfected into cells and integrated into the genome, GFP protein was expressed in the host cells. After 12 h of transfection, the cells were observed under the fluorescence microscope to evaluate transfection efficiency. Overexpression of target gene (IFITM3) was detected using RT-PCR.

### Cell treatment

The stably transfected cell lines, PBIFM3 and PBv, were divided respectively into 2 groups: control and LPS. In LPS groups, cells were incubated correspondingly with LPS (100 μg/mL) for 4, 8, 12 h. Cells in 0 h group were cultured in basal medium. After treatment, cells were collected to carry out target genes analysis using real-time PCR.

### cDNA synthesis and PCR

Cells were seeded in a 6 well plates at 2×10^5^ cells/well and treated with LPS at the concentration of 100 μg/mL. After 4, 8 and 12 h incubation, total RNA was extracted from cultured cells using Trizol reagent, and cDNA was prepared using a reverse transcriptase kit. PCR analysis was performed with a SYBR Green PCR Kit using a real-time fluorescence quantitative PCR instrument (Eppendorf Mastercycler eprealplex, Hamburg, Germany), with a reaction volume of 20 μL. The PCR program was as follows: 95 °C 15 s, 60 °C 20 s, and 72 °C 20 s, for 40 cycles.

Swine target gene mRNA specific primers are listed in Table 1. The synthesis of all primers was carried out by Shanghai Sangon Co. Ltd. (Shanghai, China). Each sample was analyzed in triplicate, and RT-PCR results were analyzed and evaluated using the relative quantity Ct method. Melting curve analysis was used to confirm the specificity of primers. The expression of the target genes were normalized as the ratio of target gene/GAPDH mRNA.

### Statistical analysis

All results are presented as the mean ± standard deviation. SPSS (Statistical Package for the Social Sciences) statistical software (version 13.0) was used to analyze data (IBM SPSS Inc., Armonk, NY, USA). One-way analysis of variance (ANOVA) was employed to analyze the differences among groups. *P*-values less than 0.05 were taken to indicate statistically significant differences.

## Abbreviations

IFNs: interferons
IFITM3: Interferon inducible trans-membrane protein 3
TLR4: toll like receptor 4
LPS: lipopolysaccharide
PB: Piggyback
GFP: green fluorescent protein
MyD88: myeloid differentiation 88
NFκB: Nuclear factor-κB
p38 MAPK: p38 mitogen-activated protein kinases
TNFα: tumor necrosis factor alpha
TBK1: TANK-binding kinase 1
bp: base pairs
IL-1β: interleukin 1 beta
CDS: coding sequence
